# Prevention first – modelling evidence-based prevention with the dental team for children in England

**DOI:** 10.1038/s41415-026-9626-6

**Published:** 2026-05-22

**Authors:** Victoria Niven, Immanuel Sakkilian, Paul R. Harper, Jennifer E. Gallagher

**Affiliations:** 026419683541247509603 Faculty of Dentistry, Oral and Craniofacial Sciences, King´s College London, Bessemer Rd, London, United Kingdom; 604007793209712937680 School of Mathematics, Cardiff University, Cardiff, United Kingdom; 757567330933385940859 Faculty of Dentistry, Oral and Craniofacial Sciences, King´s College London, Bessemer Rd, London, UK; Engagement and Service, King´s College London, UK; International Association of Dental Research, Virginia, USA

## Abstract

**Background** The United Kingdom has a diverse, professionalised dental workforce. Dental caries remains a prevalent disease, with significant inequalities. New ways of using our dental care professionals need to be considered in delivering preventive care to all patients.

**Methods** Data sources for an operational research optimisation model included child (under 18 years) population demography; National Health Service (NHS) data; epidemiological data on dental caries experience from three national surveys; and preventive guidance identified from Delivering Better Oral Health V4. Data for care delivery included timings were informed by previous research and NHS working patterns. The Linear Programming model was developed for six different dental skill mix scenarios.

**Results** Skill mix utilisation reduces time and workforce numbers, when compared with a single member of the dental team providing the preventive treatment plan. The workforce capacity ranged from 409–7,991 for dentists, 6,002–17,500 for dental therapists and 979–4,083 for extended duties dental nurses. Modelling suggests that a combination of all three dental team members provides the most efficient skill mix for this care delivery.

**Conclusion** The development of this operational research model suggests the potential for skill mix in delivering evidence-informed, risk-based prevention of oral and dental disease in children in England in dental settings and beyond.

**Supplementary Information:**

Zusatzmaterial online: Zu diesem Beitrag sind unter 10.1038/s41415-026-9626-6 für autorisierte Leser zusätzliche Dateien abrufbar.

## Key points

- As evidence-based prevention and skill mix utilisation become a dental healthcare focus, it is important we consider novel ways of informing decision-making.
- The development of this operational research model suggests the potential for skill mix in delivering evidence-informed, risk-based prevention of oral and dental disease in children in England.
- These workforce capacity requirements can inform workforce planning and strategic decision making nationally.

**Supplementary Information:**

Zusatzmaterial online: Zu diesem Beitrag sind unter 10.1038/s41415-026-9626-6 für autorisierte Leser zusätzliche Dateien abrufbar.

## Introduction

Over the last few decades, we have witnessed a global paradigm shift in health and care, involving a re-orientation towards prevention.^1^^,^^2^^,^^3^^,^^4^ In the United Kingdom (UK), the government's 2025 mandate to National Health Service (NHS) England includes ‘prioritising prevention over treatment', one of the three strategic shifts of the developing ten year health plan, and embracing contemporary evidence in the delivery of dental care.^5^^,^^6^

The UK has a diverse, professionalised dental workforce, with over 80% of registered dental professionals based in England.^7^ Of the General Dental Council (GDC) registrants in England in 2024, 46,309 (36.9%) were dentists, 7,313 (5.8%) dental therapists (DThs), 10,460 (8.3%) dental hygienists (DHs) and 64,956 (51.8%) dental nurses. Dental nurses can extend their scope of practice by training in additional skills and deliver key elements of preventive oral healthcare as extended duties dental nurses (EDDNs).^8^ Since 2013, patients can directly access dental hygienists and dental therapists (DH/DThs)^9^, albeit only in the private sector initially. Legislative change in 2024 has provided greater autonomy for them to supply and administer certain medications directly,^10^ and provide frontline NHS care.^11^ Delivering better oral health: an evidence-based toolkit for prevention is a publicly accessible resource for dental teams,^12^ and recognised as good practice internationally to guide preventive care.^13^ Furthermore, it provides risk-based preventive guidance.

Despite being a largely preventable disease,^14^ recent evidence suggests that 29.3% of five-year-olds had visible caries in 2024.^15^ The Office for Health Improvement and Disparities has stated the ambition that every child should grow up free of tooth decay, to help give them the best start in life.^16^ Inequalities persist, with children from the most deprived areas being almost three times as likely to have experience of dentinal decay. As we endeavour to give every child the best start in life^17^^,^^18^ and ‘make every contact count'^19^ to embed lifelong health supportive behaviours, it is recognised those at greater risk may require a greater level of dental care.

There is an opportunity to use the diverse skilled dental workforce to improve the quality and efficiency of preventive care we provide our patients, and potentially increase professional satisfaction.^20^ It has been identified that ‘greater use of ‘skill mix' models could have a substantial role in the future, as dentistry moves from a ‘cure' to a ‘care' culture ^21^ yet there is still limited research on implementation in the oral health workforce.^22^ Research exploring workforce modelling in the delivery of dental care has identified the potential to use the skills of dental care professionals (DCPs), especially among target or vulnerable populations.^23^^,^^24^ To date, there has been little examination of dental skill mix to incorporate evidence-based preventive care to a population of children.

## Objectives

- To build an operational research model for the use of different skill mix within the dental team to deliver evidence-informed, risk-based prevention in children in England
- To determine workforce capacity requirements to inform workforce planning and strategic decision making nationally.

## Methods

In order to build a risk-based model of care using dental skill mix and evidence-based prevention in needs-led operational research modelling the methodology of linear programming optimisation model was implemented.^25^ The design used in previous skill mix optimisation research was adopted, where both a need/demand model and a workforce/supply scenario produce skill mix optimisation.^23^ Five data inputs were required to inform the workforce requirements for four different scenarios (see Table 1).

**Table 1:** (a) Child population of England 2022 and (b) predicted future child population

**(a) Age**	**Number**	**Proportion (%)**	**(b) Year**	**Predicted child population**
0-4	3,237,500	26	2023	12,059,255
5-9	3,542,074	29	2030	11,643,646
10-14	3,436,207	28	2040	10,807,953
**0-17**	**12,097,402**	**100**	2050	11,121,906
Source: (a) NHS Dental Services, NHS Business Services Authority (BSA), NHS Digital, NHS Dental Statistics 2021-22 (b) Office for National Statistics, 2023, National Population Projections				

### Data for evidence-based preventative need

#### Child population

**Fig. 1:**
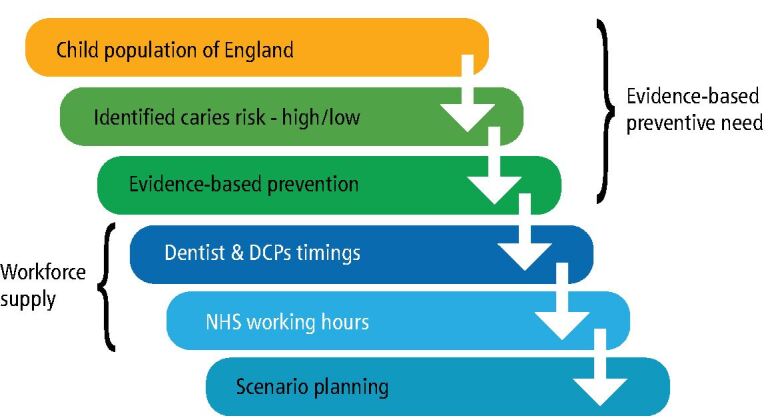
Data sources required for Linear Programming Optimisation Model

In this paper the term ‘children' refers to children and young people (under 18 years). Demographic data regarding the child population of England 2022, and predicted projections up to the year 2050, were sourced from the Office for National statistics (see Fig. 1).^26^

#### Identified caries risk: high or low caries risk

The caries risk of child population of England for different categories was identified based on past caries experience. In this paper, ‘risk' will refer to caries risk. These age categories were used in replication of Office for National Statistics 2021 ethnicity census data.^27^ The three main sources of data to identify caries risk were National Dental Epidemiology Programme for England:^1^ oral health survey of three-year-old children 2020;^28^ the 2019 oral health survey of five-year-olds;^29^ and the child health survey 2013.^30^ The proportion of children in each age category at high risk were an average of these epidemiological results and were used as the basis for risk categorisation for the proportion of children of each age. The average risk rate for the child population of England was then calculated to be 31.02 (see online Supplementary Information 1).

#### Evidence-based preventative advice and interventions

The Delivering Better Oral Health version 4 toolkit identifies advice that dental professionals should give to the whole child population (based on age), regardless of caries risk level – universal advice.^12^ If a child is identified as higher risk for developing caries, additional preventative advice and interventions are recommended, shown in online Supplementary Information 2.

#### Dentist and dental care professionals care delivery timings

Given the scope of practice for members of the dental team (dentists, DH/DThs and EDDNs), the time it takes for professionals to undertake each element of the evidence-based preventive treatment plan, in minutes, were informed by previous research (see online Supplementary Information 2).^31^ Given that 5,706 DHs and DThs (32.7%) were dually qualified in 2024, and many current graduates are dually qualified, and the evidence of care timings was presented with DHs and DThs as one group, we have presented DHs and DThs as a combined group also (DH/DThs). These calculations were developed in line with the rationale presented in Box 1.

Box 1 Rationale for timings

- Every child in England attends an NHS dental practice (100% attendance) once a year for an oral health review once a year, and subsequent preventative care plan.
- All necessary restorative treatment can be undertaken in 30 minutes/year for high-risk children, in all age categories.
- EDDNs timings the same as DH/DThs, and EDDNs provide oral health promotion under the prescription of a dentist or a DH/DTh
- Except for clinical examination/treatment planning which will be a confirmatory examination for EDDNs – estimated two minutes
- 0-4s no radiographs and modified the timings for F varnish as applied three times a year for high risk but not for 0-year-olds (no dentition) so only 1–4-year-olds within that age group would receive F varnish. F varnish for low risk 3-4-year-olds only
- F varnish is applied three times/year high risk 5-17 year olds and twice a year to low risk
- In high-risk category 5–9-year-olds four fissure sealants will be completed (secondary first molars/6s)
- As fissure sealants are, it is assumed, only applied once per tooth in each child, the time required to provide the number of fissure sealants per age category has been divided across the number of years in each category
  - 5–9-year-olds (five years) four FS provided (9.3mins per FS)
  - 9.34/5=7.44 minutes per year
- In the higher risk category, amongst 10–17-year-olds, it was estimated that 12 fissure sealants could be completed (premolars and second and third molars/4578s in all quadrants) once over this 8year period 10–17-year-olds
  - 9.3×12/8=13.95 mins per year
  - Thus, for a dentist to undertake all elements of this care for one high risk patient in this age group, it would take them up to 90 mins (89.65 minutes)
- VBA regarding tobacco and vaping use assumed once a year 10–17-year-olds. As 39% of 11-17-year-olds are ‘never smokers'; thus, brief low risk exploration only taking 0.3mins for all DCPs.

DCPs, dental care professionals; DH, dental hygienist; DThs, dental therapists; EDDNs, extended duty dental nurses; F varnish, fluoride varnish; FS, fissure sealants; NHS, National Health Service; VBA, very brief advice.

### Data for dental team workforce requirements

#### Dentist and dental care professionals NHS working hours

Using the most recent pre-covid data for the number of hours dentists provide NHS clinical care for patients, it was identified that dentists dedicate an average of 26.7 hours a week to NHS care with an average of 4.4 weeks annual leave.^32^ From the government national careers website, dental nurses work on average a similar number of hours per week to dentist, so it was extrapolated that a similar proportion of hours would be spent providing NHS care – 26.7 hours – and would take on average 4.4 weeks annual leave per year.^33^ Based on evidence on the working patterns of dental hygienists, working approximately 25 hours a week, for DH/DThs in England the mean percentage of patients treated under NHS contract was 47.2%, thus it was assumed that 11.8 hours a week (also with 4.4 weeks annual leave) of DH/DThs working time would be dedicated to providing NHS treatment.^34^ In this model, the assumption was made that all the dentists and DCPs dedicate 100% of their NHS time to treating children.

#### Formulation

**Fig. 2:**
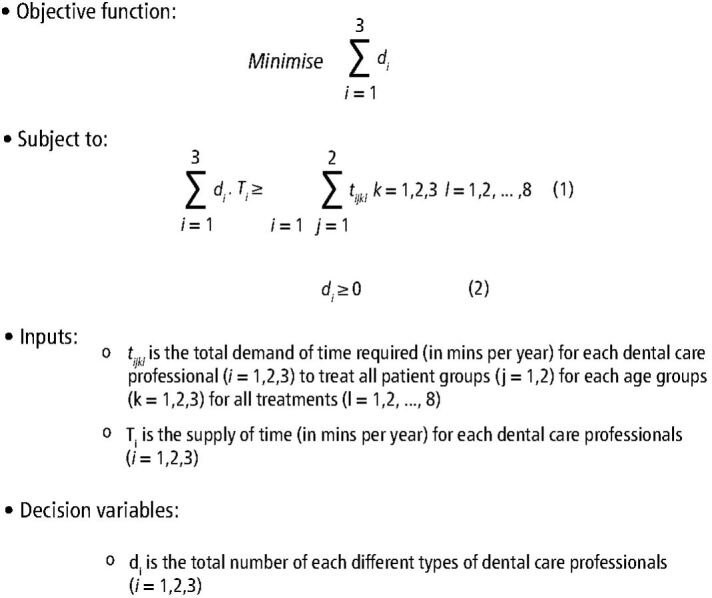
The linear programme formulation

The linear programme formulation minimises the total workforce numbers/volume used to satisfy the total time needed to treat the child patients in each group and at each risk level, given the treatment and associated timings identified (see Fig. 2). Validation and verification are important processes in operational research modelling, ensuring the model is both accurate and reliable. For model verification, we ensured that the optimisation model solution was sound and logical (for example ensuring that no constraints were violated), and for validation we to check that the results were within the expected range for both with known data (the status quo) and expert opinion for modelling future scenarios.^35^

## Results

### The scenarios

Using the data, six skill mix scenarios were explored to identify the optimal skill mix for delivery of evidence-based preventive care for the child population of England.^8^ The models are presented with increasing skill mix utilisation (see online Supplementary Information 3). For all the scenarios presented, the workforce numbers provided represent whole-time equivalent (WTE), summarised in online Supplementary Information 4.

#### No skill mix – dentists provide all preventive care

The first model identifies the number of dentists required to provide the child population of England with evidence-based preventive care for the year. Given the risk rate calculation of 31.02%, in 2023 it can be estimated 7,991 dentists would be required to deliver this care to the child population of England. Incorporating future child population projections, and adjusting for risk rate changes, if the caries risk rate reduces due to preventive care provision by 2050 to 16.56% (one standard deviation lower than 31.02%), the dentists required would reduce to 6,368 (See online Supplementarty Information 5).

#### No skill mix – dental hygienists and dental therapists provide all preventive care

To deliver the caries preventive care to the child population of England, a greater number of DH/DThs would be required, compared with dentists (see online Supplementarty Information 5). This is WTE – each DH/DThs dedicating all their NHS clinical time (which is much less than dentists or EDDNs) to seeing only child patients. In 2023, given the risk rate calculation of 31.02%, it can be estimated 17,500 DH/DThs would be required to deliver care.

#### Moderate skill mix – dental hygienists and dental therapists and extended duties dental nurses

When DH/DThs provide the initial exam to all child patients, and the fissure sealants and treatment in high-risk patients, then delegating all remaining elements of the preventive care plan to EDDNs. When only DH/DThs and EDDNs deliver the preventive treatment plan, and no dentists are involved in the care plan or delivery, notably fewer DH/DThs are required than in scenario b) with the inclusion of EDDNs (see online Supplementarty Information 5). In 2023, given the risk rate calculation of 31.02%, it can be estimated 8,502 DH/DThs and 4,083 EDDNs – a total combined workforce of 12,585 – would be required to deliver preventive care.

#### Intermediate skill mix – dentists, dental hygienists, dental therapists and extended duties dental nurses

In this scenario, the dentist performs the initial examination and treatment plan and then all elements of the care plan are delivered by DH/DThs, except the fluoride varnish application delivered by the EDDNs. This skill mix requires a greater total workforce to deliver the treatment plan than the minimum skill mix model (see online Supplementarty Information 5). In 2023, given the risk rate calculation of 31.02%, it can be estimated 1,602 dentists, 15,169 DH/DThs and 979 EDDNs – a total combined workforce of 17,750 – would be required to deliver preventive care.

#### Combination skill mix – proportional skill mix

This model reflects the complexity of needs of dental patients. In this scenario, dentists will provide all the preventive care for 10% of child patients (high risk), with DH/DThs and EDDNs providing the preventive care plans for the remaining high caries risk child patients, and the lower caries risk group for each age group (see online Supplementarty Information 5). In 2023, given the risk rate calculation of 31.02%, it can be estimated 409 dentists, 7,902 DH/DThs and 3,940 EDDNs – a total combined workforce of 12,251 – would be required to deliver care.

#### Maximum skill mix

Maximum care delegation is reflected in this scenario, with EDDNs providing all elements of the treatment plan within their scope of practice, DH/DThs providing fissure sealants and restorations following an oral health assessment for the all the patients by a dentist (see online Supplementarty Information 5). In 2023, given the risk rate calculation of 31.02%, it can be estimated 1,602 dentists, 6,002 DH/DThs and 4,083 EDDNs – a total combined workforce of 11,687 – would be required to deliver preventive care.

## Discussion

The models presented in this paper demonstrate options for a shift from parallel practice to teamworking in the delivery of oral care in general dental practice. Maximising the potential of the wider dental team has the potential to facilitate effective care of our patient population, with delegation of care to DH/DThs being shown to be positively experienced by dental patients.^36^^,^^37^ This novel study demonstrates the potential opportunities for the dental team to deliver evidence-based preventive oral care to the child population of England, especially at a time of ‘crisis to access'.^38^ From the linear programming optimisation models, which incorporate the different NHS working patterns of the dental team, the scenario which requires the lowest workforce is when all elements of preventive care are delivered by dentist (n = 7,991). In 2024, 46,362 dentists registered with the GDC.^39^ Of the more than 35,000 registered dentists in England, just over 24,000 delivered some (although maybe not exclusively) dental care for the NHS in 2023/24.^40^ In this model, therefore, one third of the dentist workforce of England would be required to dedicate 100% of their NHS clinical working time to deliver evidence-based preventive care plans to the child population of England. Approximately 22% of England's population is under 18 years, based on the 2021 census data, and comprising nearly 27% of the NHS dental patients seen in the 12 months up to March 2024^41^^,^^42^ shared across the workforce, represented in the scenarios of the model.

However, when introducing skill mix to the delivery of preventive oral care plans, the maximum skill mix scenario requires the lowest the lowest workforce number (n = 11,687). The highest workforce numbers are required for the intermediate skill mix model (n = 17,750). These findings suggest that effective skill mix, using the dental team for the tasks at which they are most skilled, has the potential to optimise the efficient delivery of preventive oral care.

As the number of dentists with NHS activity has fallen from 43.4 per 1000,000 in 2012/12 to 42.3 per 100,000 in 2022/23, and their NHS commitment reduces, the need to explore alternative methods of care delivery has never been more important.^11^^,^^43^Although the government has stated its ambition for everyone in England to be able to access an NHS dentist, it might be more reasonable to strive to increase access to dental care delivered by an appropriately skilled dental care professional.^44^

Planning the future oral health workforce is heavily reliant on quality data being available for models.^45^ The data inputs for the models are contemporaneous and can be used reflexively and sustainably as new evidence emerges. In this model, we presented the dental hygienists and dental therapists as a combined group. We recognise that there are still many GDC registered dental hygienists, for whom the restorative treatments in these models are beyond their scope of practice. Dental hygienists play a key role in oral health promotion and in future modelling it will be essential we consider them separately, along with geographic regional service planning.

Projections have been incorporated in the models, given population growth estimates which allow for responsive workforce estimates. The methodologies implemented in this study with varied skill mix considerations and accounting for uncertainty and variations in parameters such as caries-risk and population, increase the potential for future planning exercises.^45^ Assumptions were made in the construct of the models, for example the timings of dental tasks undertaken by EDDNs, but these can be modified as further evidence becomes available. One scenario excludes dentists entirely from the preventive treatment plan, with DH/DThs sharing care with EDDNs. The availability of a dentist as part of the wider team, although not directly involved in certain oral care plans, would support upward referrals if required. The different care provision include the working patterns of DH/DThs that dedicate just under half of their working clinical time to the delivery of NHS care.^34^ If these working patterns change, the model parameter can be adjusted to identify the workforce requirements in each skill mix scenario. These variables, however, are dependent on the type of service provision.

The scenarios in this study present the ‘ideal' level of care provision – 100% of the child population attend dental practices at least once a year for oral health reviews and evidence based oral health promotion and caries risk-appropriate care. In 2023, only 52.7% (5.35 million) children in England had attended an NHS dentist within the maximum recommended interval of one year – children's services need to be expanded to maximise access. If only half the child population access NHS dental services, half the workforce numbers identified in these models may currently be required. Additional time inclusions could be for transfer between different team members, oral care delivery for urgent dental treatment, and more complex oral care plans. The former which could be approximated as three percent of the population require urgent dental treatment a year.^46^ When implementing task sharing for oral care administrative complications of multiple appointment scheduling and clinical space requirements for dental team members to provide differing components of the care plan would need to be considered. Lean pathways (which streamline healthcare processes) would need to be embedded not only to ensure efficiency of care delivery but for patient acceptability, clinical and environmental sustainability.^47^

The models could be developed to explore adult care provision, target groups commissioning cost comparisons and workforce requirement variations at regional or local level given the unequal distribution of dentists across England, being more highly concentrated around metropolitan areas and dental schools.^48^

The workforce requirements in the model assumes that each dental team member dedicates 100% of their NHS clinical working time to the provision of care for children, which is an unlikely reality in general dental practice.^49^ Further research could investigate the opportunity to commission or appetite from dentists and/or DCPs to provide NHS general dental care exclusively to children, with the potential for flexible commissioned dedicated sessions to address child oral health inequalities.^50^ This could be supported by a new enhanced skills training for dental nurses to provide such dedicated care and potential career development opportunities to assist with retention.

Challenges persist in the fostering of skill mix as ‘the norm' in dental practice in England – although not a new concept, interprofessional collaboration in general dental practice is not common, especially compared with models of care sharing in general medical practice in the UK.^11^ ultimately it is down to services providers within the NHS and private sectors. It is imperative we consider ways to support, embed, and facilitate the delivery of alternative models of collaborative oral care to effectively, efficiently and equitably through national and local policy and commissioning to meet the developing needs of the population within the NHS, especially children and young people.

### Summary

The development of this operational research model suggests the potential for skill mix in delivering evidence-informed, risk-based prevention of oral and dental disease in children in England. These workforce capacity requirements can inform workforce planning and strategic decision making nationally.

## Supplementary Information

The proportion of children in England with caries experience and category high risk rates (PDF 60KB)

Timings for High caries risk and lower caries risk evidence-based prevention by dental team members for child population of England, by age category. (PDF 138KB)

Skill-mix scenarios (PDF 24KB)

Workforce requirements for each scenario in 2023 assuming a 31.02% high caries risk rate (PDF 91KB)

Workforce required to deliver preventive care to children in England given high caries risk rate for 2023 and future population projections. (PDF 185KB)

## Data Availability

Publicly available data were used in this research.
