# Supplementary material for: Prevention first – modelling evidence-based prevention with the dental team for children in England
Source: Br Dent J. 2026 May 22;240(10):681–6. doi: 10.1038/s41415-026-9626-6 (PMC13197221; doi:10.1038/s41415-026-9626-6)
Supplement: Supplementary file 1 — The proportion of children in England with caries experience and category high risk rates (PDF 60KB) [file 41415_2026_9626_MOESM1_ESM.pdf]

Table 2 The proportion of children in England with caries experience and category high risk rates

Children age in years	Percentage with caries
3 year-olds[1]	10.7
5 year-olds[2]	23.4
8 year-olds[3]	45
12 year-olds[3]	32
15 year-olds[3]	44
Average	31.0

Children age in years	% Caries High Risk	% Caries Low Risk
0-4	10.7	89.3
5-9	34.2	65.8
10-17	38	62
Average Rate	31.02	68.98

1 National Dental Epidemiology Programme for England: oral health survey of 3-year-old children 2020
2 National Dental Epidemiology Programme for England: oral health survey of 5-year old children 2019
3 Child Dental Health Survey, 2013
Gallagher, J. 2022. Creativity, confidence and the courage to change: the future dental workforce – part two. *Faculty Dental Journal,* 13**,** 142-149; Gallagher, J. E. 2019. The Future Oral and Dental Workforce for England: Liberating the future workforce to serve the population across the Life Course. *Advancing Dental Care*. London: Health Education England.
