# Supplementary material for: Prevention first – modelling evidence-based prevention with the dental team for children in England
Source: Br Dent J. 2026 May 22;240(10):681–6. doi: 10.1038/s41415-026-9626-6 (PMC13197221; doi:10.1038/s41415-026-9626-6)
Supplement: Supplementary file 2 — Timings for High caries risk and lower caries risk evidence-based prevention by dental team members for child population of England, by age category. (PDF 138KB) [file 41415_2026_9626_MOESM2_ESM.pdf]

Table 3a Timings for High caries risk and lower caries risk evidence-based prevention by dental team members for child population of England, by age category.

Age Band	High Caries Risk Prevention				Lower Caries Risk Prevention			
	Preventative Care Plan	Treatment Timings for Dentists in minutes	Treatments Timings for DH/DThs in minutes	Treatments Timings for EDDNs in minutes	Preventative Care Plan	Treatment Timings for Dentists in minutes	Treatments Timings for DH/DThs in minutes	Treatments Timings for EDDNs in minutes
0-4	Clinical examination and treatment planning	10	10	2	Clinical examination and treatment planning	10	10	2
	Health advice: diet	5	5.6	5.6	Health advice: diet	5	5.6	5.6
	Health advice: oral hygiene instruction	5	6.8	6.8	Health advice: oral hygiene instruction (fluoride toothpaste + tooth brushing)	5	6.8	6.8
	Apply fluoride varnish (2.26% NaF) to teeth 3 times a year (ages 1-4 yo only)	15.36	7.2	7.2	Apply fluoride varnish (2.26% NaF) to teeth 2 times a year (ages 3 & 4yo only)	5.12	2.4	2.4
	Treatment	30	30	0	N/A	0	0	0
5-9	Clinical examination and treatment planning	10	10	2	Clinical examination and treatment planning	10	10	2
	Radiograph(s) one/multiple	4.4	6.4	6.4	Radiograph(s) one/multiple	4.4	6.4	6.4
	Health advice: diet	5	5.6	5.6	Health advice: diet	5	5.6	5.6
	Health advice: oral hygiene instruction	5	6.8	6.8	Health advice: oral hygiene instruction (fluoride toothpaste + tooth brushing)	5	6.8	6.8
	Apply fluoride varnish (2.26% NaF) to teeth 3 times a year.	19.2	9	9	Apply fluoride varnish (2.26% NaF) to teeth 2 times a year. (6.4X2)	12.8	6	6

	Fissure sealant application X4	7.44	12.7	0	N/A	0	0	0
	Treatment	30	30	0	N/A	0	0	0
10-17	Clinical examination and treatment planning	10	10	2	Clinical examination and treatment planning	10	10	2
	Radiograph(s) one/multiple	4.4	6.4	6.4	Radiograph(s) one/multiple	4.4	6.4	6.4
	Health advice: diet	5	5.6	5.6	Health advice: diet	5	5.6	5.6
	Health advice: oral hygiene instruction	5	6.8	6.8	Health advice: oral hygiene instruction (fluoride toothpaste + tooth brushing)	5	6.8	6.8
	Advice on methods of quitting (Smokers) (9% of people in this age group need tobacco advice)	2.1	4.1	4.1	Very brief questioning about smoking status (Non-smokers)	0.3	0.3	0.3
	Apply fluoride varnish (2.26% NaF) to teeth 3 times a year.	19.2	9	9	Apply fluoride varnish (2.26% NaF) to teeth 2 times a year. (6.4X2)	12.8	6	6
	Fissure sealant application X 12	13.95	19.05	0	N/A	0	0	0
	Treatment	30	30	0	N/A	0	0	0
