# Supplementary material for: Prevention first – modelling evidence-based prevention with the dental team for children in England
Source: Br Dent J. 2026 May 22;240(10):681–6. doi: 10.1038/s41415-026-9626-6 (PMC13197221; doi:10.1038/s41415-026-9626-6)
Supplement: Supplementary file 3 — Skill-mix scenarios (PDF 24KB) [file 41415_2026_9626_MOESM3_ESM.pdf]

Table 4 Skill-mix scenarios

<table>
<tr><th>Scenario</th><th>Skill mix</th><th colspan="6">Dental Team Members</th></tr>
<tr><td>a</td><td>No skill mix</td><td colspan="6">Dentist</td></tr>
<tr><td>b</td><td>No skill mix</td><td colspan="6">DH/DThs</td></tr>
<tr><td>c</td><td>Moderate skill mix</td><td colspan="3">DH/DThs</td><td colspan="3">EDDN full scope</td></tr>
<tr><td>d</td><td>Intermediate skill mix</td><td colspan="2">Dentist exam</td><td colspan="2">DH/DThs remaining elements</td><td colspan="2">EDDN fluoride varnish only</td></tr>
<tr><td>e</td><td>Combination skill mix</td><td colspan="2">Dentist 10%</td><td colspan="2">DH/DThs remaining elements</td><td colspan="2">EDDN full scope</td></tr>
<tr><td>f</td><td>Maximum skill mix</td><td colspan="2">Dentist exam</td><td colspan="2">DH/DThs remaining elements</td><td colspan="2">EDDN full scope</td></tr>
</table>
