# Supplementary material for: Prevention first – modelling evidence-based prevention with the dental team for children in England
Source: Br Dent J. 2026 May 22;240(10):681–6. doi: 10.1038/s41415-026-9626-6 (PMC13197221; doi:10.1038/s41415-026-9626-6)
Supplement: Supplementary file 4 — Workforce requirements for each scenario in 2023 assuming a 31.02% high caries risk rate (PDF 91KB) [file 41415_2026_9626_MOESM4_ESM.pdf]

Table 6 Workforce requirements for each scenario in 2023 assuming a 31.02% high caries risk rate

Scenario	Dentists	DH/DThs	EDDNs	TOTAL
a	7,991	-	-	7,991
b	-	17,500	-	17,500
c	-	8,502	4,083	12,585
d	1,602	15,169	979	17,750
e	409	7,902	3,940	12,251
f	1,602	6,002	4,083	11,687
