# Supplementary material for: Prevention first – modelling evidence-based prevention with the dental team for children in England
Source: Br Dent J. 2026 May 22;240(10):681–6. doi: 10.1038/s41415-026-9626-6 (PMC13197221; doi:10.1038/s41415-026-9626-6)
Supplement: Supplementary file 5 — Workforce required to deliver preventive care to children in England given high caries risk rate for 2023 and future population projections. (PDF 185KB) [file 41415_2026_9626_MOESM5_ESM.pdf]

Table 5 Workforce required to deliver preventative care to children in England given high caries risk rate for 2023 and future population projections.

a

Dentists					
Year	High Caries Risk Rate				
	2.10%	16.56%	31.02%	45.48%	59.94%
2023	5,819	6,905	**7,991**	9,077	10,162
2030	5,619	6,667	7,715	8,764	9,812
2040	5,216	6,189	7,162	8,135	9,108
2050	5,367	6,368	7,370	8,371	9,372

b

DH/DThs					
Year	High Caries Risk Rate				
	2.10%	16.56%	31.02%	45.48%	59.94%
2023	12,433	14,966	**17,500**	20,033	22,567
2030	12,004	14,450	16,897	19,343	21,789
2040	11,143	13,413	15,684	17,955	20,225
2050	11,466	13,803	16,140	18,476	20,813

c

DH/DThs					
Year	High Caries Risk Rate				
	2.10%	16.56%	31.02%	45.48%	59.94%
2023	3,954	6,228	8,502	10,776	13,050
2030	3,818	6,013	8,209	10,405	12,600
2040	3,544	5,582	7,620	9,658	11,696
2050	3,647	5,744	7,841	9,938	12,035
EDDNs					
Year	High Caries Risk Rate				
	2.10%	16.56%	31.02%	45.48%	59.94%
2023	3,865	3,974	4,083	4,192	4,301
2030	3,732	3,837	3,942	4,047	4,153
2040	3,464	3,561	3,659	3,757	3,855
2050	3,564	3,665	3,765	3,866	3,967
**TOTAL 2023**	7,819	10,202	**12,585**	14,968	17,351

d	Dentists				
Year	High Caries Risk Rate				
	2.10%	16.56%	31.02%	45.48%	59.94%
2023	1,602	1,602	1,602	1,602	1,602
2030	1,546	1,546	1,546	1,546	1,546
2040	1,435	1,435	1,435	1,435	1,435
2050	1,477	1,477	1,477	1,477	1,477
	DH/DThs				
Year	High Caries Risk Rate				
	2.10%	16.56%	31.02%	45.48%	59.94%
2023	10,447	12,808	15,169	17,530	19,891
2030	10,087	12,367	14,646	16,926	19,205
2040	9,363	11,479	13,595	15,711	17,827
2050	9,635	11,813	13,990	16,167	18,345
	EDDNs				
Year	High Caries Risk Rate				
	2.10%	16.56%	31.02%	45.48%	59.94%
2023	834	906	979	1,051	1,124
2030	805	875	945	1,015	1,085
2040	747	812	877	942	1,007
2050	769	836	903	970	1,036
**TOTAL 2023**	12,883	15,316	**17,750**	20,183	22,617

e

Dentists					
Year	High Caries Risk Rate				
	2.10%	16.56%	31.02%	45.48%	59.94%
2023	28	218	409	599	789
2030	27	211	394	578	762
2040	25	195	366	537	708
2050	26	201	377	552	728
DH/DThs					
Year	High Caries Risk Rate				
	2.10%	16.56%	31.02%	45.48%	59.94%
2023	3,914	5,908	7,902	9,896	11,890
2030	3,779	5,704	7,629	9,555	11,480
2040	3,508	5,295	7,082	8,869	10,656
2050	3,609	5,449	7,288	9,127	10,966
EDDNs					
Year	High Caries Risk Rate				
	2.10%	16.56%	31.02%	45.48%	59.94%
2023	3,855	3,898	3,940	3,983	4,025
2030	3,722	3,763	3,804	3,845	3,886
2040	3,455	3,493	3,531	3,569	3,607
2050	3,555	3,595	3,634	3,673	3,712
**TOTAL 2023**	7,797	10,024	**12,251**	14,478	16,704

f

Dentists					
Year	High Caries Risk Rate				
	2.10%	16.56%	31.02%	45.48%	59.94%
2023	1,602	1,602	1,602	1,602	1,602
2030	1,546	1,546	1,546	1,546	1,546
2040	1,435	1,435	1,435	1,435	1,435
2050	1,477	1,477	1,477	1,477	1,477
DH/DThs					
Year	High Caries Risk Rate				
	2.10%	16.56%	31.02%	45.48%	59.94%
2023	406	3,204	6,002	8,800	11,598
2030	392	3,094	5,795	8,497	11,198
2040	364	2,872	5,379	7,887	10,395
2050	375	2,955	5,536	8,116	10,697
EDDNs					
Year	High Caries Risk Rate				
	2.10%	16.56%	31.02%	45.48%	59.94%
2023	3,865	3,974	4,083	4,192	4,301
2030	3,732	3,837	3,942	4,047	4,153
2040	3,464	3,561	3,659	3,757	3,855
2050	3,564	3,665	3,765	3,866	3,967
**TOTAL 2023**	5,873	8,780	**11,687**	14,594	17,501
